# Long-term outcomes after chest wall resection and repair with titanium bars and sternal plates

**DOI:** 10.3389/fsurg.2022.950177

**Published:** 2022-09-07

**Authors:** Hugo Clermidy, Guillaume Fadel, Alexandra De Lemos, Pauline Pradere, Delphine Mitilian, Antoine Girault, Jean-Baptiste Menager, Dominique Fabre, Sacha Mussot, Nicolas Leymarie, Elie Fadel, Olaf Mercier

**Affiliations:** ^1^ Department of Thoracic and Vascular Surgery and Heart-Lung Transplantation, Université Paris-Saclay, International Center for Thoracic Cancers, Marie-Lannelongue Hospital, GHPSJ, Le Plessis Robinson, France; ^2^ Department of Pneumology, Marie-Lannelongue Hospital, Groupe Hospitalier Paris Saint-Joseph, Le Plessis Robinson, France; ^3^ Department of Plastic and Reconstructive Surgery, Gustave Roussy, Villejuif, France

**Keywords:** chest wall resection, titanium bars, infection, fracture, long term

## Abstract

**Objectives:**

En-bloc complete resection remains the treatment of choice for localized chest wall (CW) tumors. Titanium bars reconstruction demonstrated encouraging results with satisfactory early outcomes. However, long-term outcomes remain under-reported. The purpose of this study is to evaluate long-term outcomes after CW resection and repair with titanium devices.

**Methods:**

From June 2012 to December 2018, we retrospectively reviewed all patients with CW tumors who underwent surgical resection and repair using titanium. Long-term outcomes were assessed.

**Results:**

We identified 87 patients who underwent CW tumor resections and titanium reconstruction. Sixty-eight patients were included in the study (excluding benign tumors, Pancoast tumors, palliative surgeries, or clavicle reconstruction). There were 29 sarcomas, 20 isolated CW metastases, eight lung cancers, four breast cancers, three thymic malignancies, two sarcomatoid mesothelioma, and one desmoid tumor. Complete resection was achieved in 64 patients (94%), while R1 resection in four patients (6%). Resection involved one rib in two patients, two ribs in thirteen, three ribs in eighteen, four ribs in nine, five ribs in two, seven ribs in one, partial sternum in fifteen, and full sternum in sixteen patients. No patient experienced flail chest. The 1-year, 3-year, and 5-year overall survival rates and disease-free survivals were 82.3%, 61.4%,57.3%, and 67.6%,57.3%,52.6%, respectively. Surgical site infection occurred in 18% (*n* = 12) of cases. Eleven of twelve patients had an early infection (<1 year), which required material removal in six patients. Asymptomatic connector unsealing occurred in 6% (*n* = 4), with only one re-intervention. Titanium allergy has never been reported. Chronic chest pain (lasting more than 3 months after surgery, with daily use of pain killer) was reported in 24% of patients.

**Conclusion:**

CW resections with titanium reconstruction are associated with long-term survivors. Titanium devices were safe, reliable, and achieved satisfactory oncological results with low morbidity and implant-related complication rates.

## 1 Introduction

Chest wall (CW) tumors are a very uncommon heterogeneous group of thoracic tumors. The prevalence of primary malignant bone and soft tissue tumors is reported from 1% to 2% of all thoracic neoplasms whereas CW metastasis or involvement from lung or breast cancers are more frequent (1). It is well-established that complete *en-bloc* resection with free surgical margins is the cornerstone to achieving the best oncological outcomes (2, 3). Surgery conducted by a multidisciplinary team including thoracic and plastic surgeons allows for more extended surgery, which could become mandatory for large tumors. Parallel to the extent of the resection, the actual challenge is to maintain the CW integrity and stability, to ensure the protection of intrathoracic organs and adequate respiratory function, as well as acceptable cosmetic results regardless of the dimension of the tumor. A wide range of synthetic and autogenous materials have been used in CW reconstruction. The choice of material usually depends on the location and extent of the defect, together with the experience of the surgical team. However, the type of material used for reconstruction remains controversial. Historically, large thoracic defects used to be repaired with polytetrafluoroethylene (PTFE), polypropylene with or without methylmethacrylate mesh, or autologous myocutaneous flaps. Without rigid reconstruction, thoracic insufficiency syndrome with CW distorted by curve rotation, volume depletion, and transient paradoxical respiration has been described (4). For a decade, titanium bars and sternal plates have gradually been used in CW reconstruction, with promising early outcomes (5–8). Titanium, which is biologically inert and biocompatible material, brings a true benefit compared to prosthetic meshes which have a lack of rigidity and an infection rate reported at 20% (9). It is also adaptable to a wide variety of CW defects, allowing to recreate the anatomical and physiological appearance of the thoracic cage. In case of a soft tissue defect, muscle or musculocutaneous flaps (mainly pectoralis major or latissimus dorsi flaps) can be used for covering prosthetic material. However, this new type of reconstruction leads to new titanium-related complications. Implant failures such as bars and connectors fracture are seen with the STRATOS™ titanium system (Strasbourg Thorax Osteosyntheses System, MedXpert GmbH, Heitersheim, Germany) (10, 11). Metal allergy is also a rare complication described in pectus repair (12, 13). Long-term results are unknown especially with pain and quality of life, and oncological outcomes. Only one study about the long-term outcome of patients in whom these bars were implanted, including mainly traumatic CW stabilization, has been published (7). The objectives of this study are to present our experience of CW resection and reconstruction with a new osteosynthesis system (Thorib® titanium bars or Trionyx® sternal plate) during surgical resection of CW malignancies and to evaluate long-term outcomes after this procedure.

## 2 Materials and methods

The study was approved by our institutional review board and the Institutional Review Board of the French Society of Thoracic and Cardiovascular Surgery. In accordance with French law on retrospective clinical studies of anonymized data, informed consent was not required. The results of this study were reported according to the strengthening the reporting of observational studies in epidemiology (STROBE) statement (14).

### 2.1 Patients

**Figure 1:**
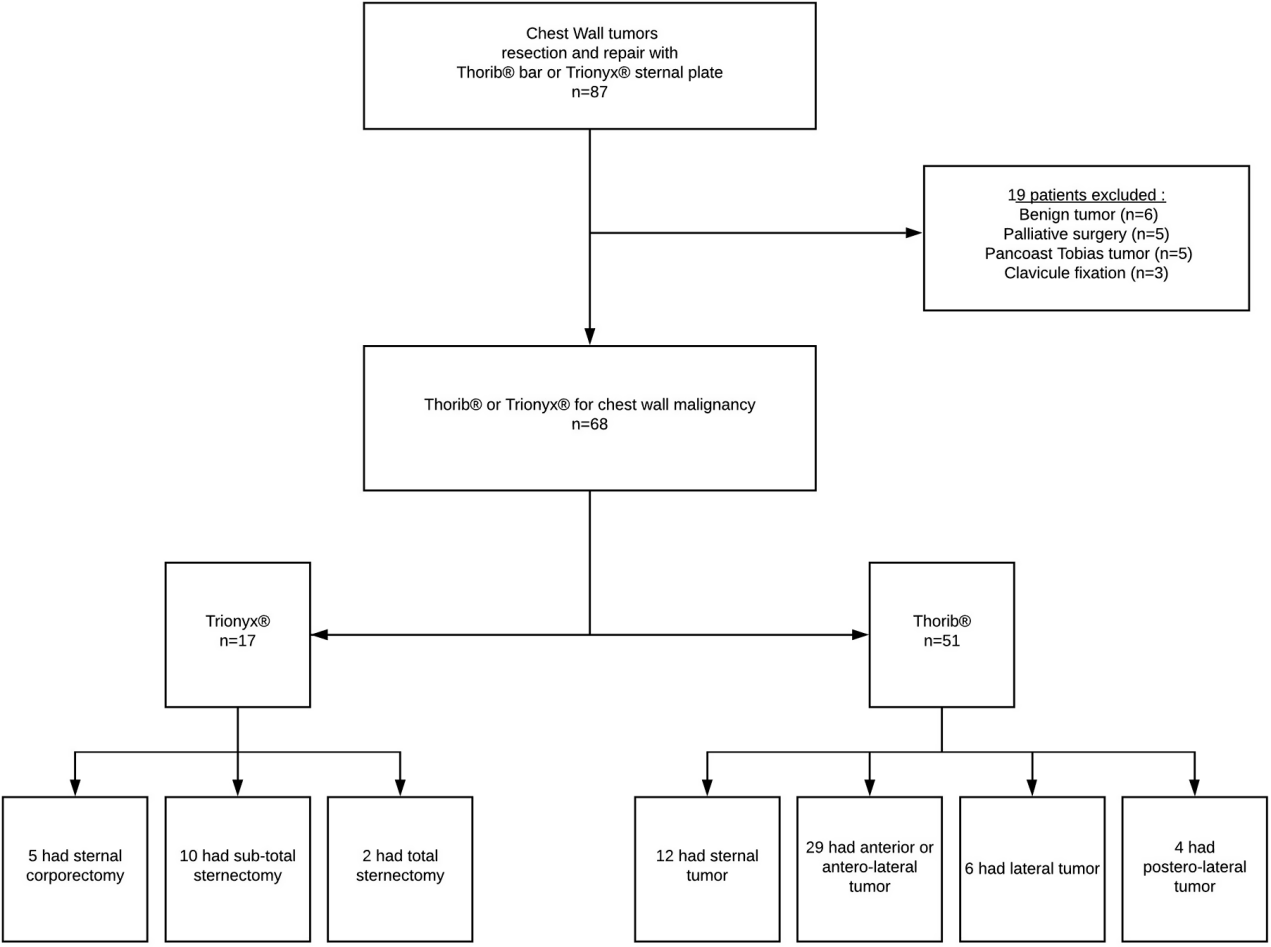
Flow chart of the study.

From June 2012 to December 2018, we reviewed all patients with CW tumors who underwent surgical rib and/or sternal resection and repair with the Thorib® or Trionyx® system (Neurofrance Implants, La Ville au Clerc, France) in the Marie Lannelongue Hospital. During this period, congenital CW deformities (as pectus excavatum or carinatum) and thoracic trauma were not included, and benign tumors, palliative surgery, and Pancoast tumors were excluded. Contraindications to curative surgical resection of CW tumors were other metastasis than the CW and N2/N3 carcinoma as determined by the preoperative workup (Figure 1).

All patients had suspected diagnosis on computed tomography (CT), carried out for pain, thoracic mass, or the extension of the initial tumor. A magnetic resonance imaging (MRI) was systematically performed to evaluate the medullary invasion for sternal tumors. For other tumors, MRI was performed to specify the extent of the CW, pleural, and mediastinal involvement. In all cases, a positron emission tomography (PET)/CT scan confirmed a localized disease to the CW with no distant metastases. The preoperative pathologic diagnosis was obtained either by CT-guided percutaneous biopsy or surgical biopsy. Cardio-respiratory function evaluation including electrocardiogram, echocardiogram, ventilation/perfusion scan, spirometry with plethysmography, and measurement of diffusing capacity of the lung for carbon monoxide (DLCO) was performed preoperatively. Diabetes, smoking history, pack years, and body mass index (BMI) were noted. All cases were discussed in a specific tumor board before surgery.

### 2.2 Surgical technique

#### 2.2.1 Chest wall resection

To isolate primary or secondary CW tumors, a direct approach focused on the lesion was performed, with skin excision of the prior biopsy. Postero-lateral thoracotomy was preferred for non-small cell lung cancer (NSCLC) infiltrating the CW. Median sternotomy incision was performed for mediastinal tumors and sternal tumors. Resection of the involved ribs with the related intercostal muscles included one segment of the intact rib above and below the gross margin of the tumor, and if possible, at least 3 cm laterally. If the primitive was NSCLC, lobectomy was associated with the parietectomy without any extra-pleural dissection. For other cases, if there were pulmonary adherences, wedge resection was performed for an en-bloc resection. When the sternum was involved, adjacent sternocostal cartilages were removed and partial or complete sternectomy was performed. A mastectomy was associated with primary breast cancer invading the CW. All other invaded structures were resected to achieve R0 procedure. Intrathoracic exploration was always performed in order to eliminate pleural extension before removing CW.

#### 2.2.2 Chest wall reconstruction

##### 2.2.2.1 Ribs reconstruction

**Figure 2:**
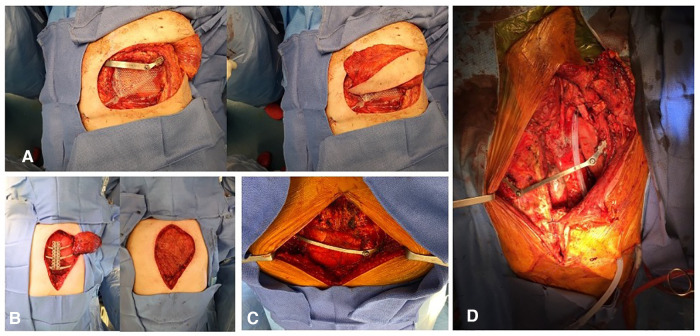
Operative field pictures showing: (**A**) Thorib® bar for lateral chest wall reconstruction with vicryl mesh interposition and pedicled homolateral latissimus dorsi flap. (**B**) Trionyx® sternal plate reconstruction after total sternectomy covered by homolateral latissimus dorsi pedicled flap. (**C**) Thorib® bar reconstruction after manubrial resection. (**D**) Thorib® bar crossing the midline and attached to the contralateral rib.

**Figure 3:**
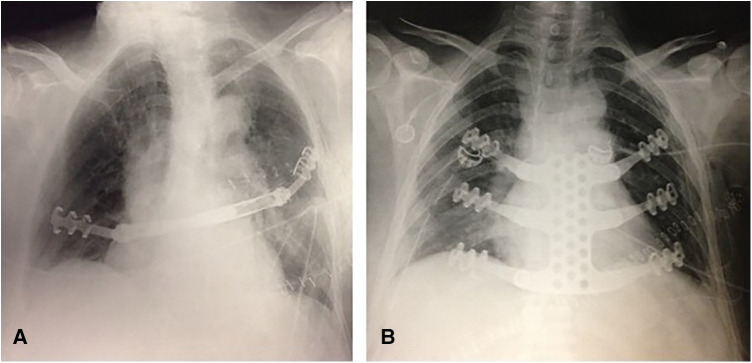
Postoperative chest x-rays after (**A**) Thorib® bar interposition and (**B**) Trionyx® sternal reconstruction.

All patients required CW reconstruction due to the removal of ≥1 anterolateral rib. Posterior and apical CW defects resection did not require prosthetic reconstruction for functional reasons because of the natural parietal suspension provided by the sternum, scapula, clavicula, and attached wide muscles of the thorax. However, defects that extend down to the fifth and sixth ribs may cause the impaction of the tip of the scapula, which is a source of discomfort. Reconstruction should be discussed for posterior resection larger than 10 cm. For anterolateral CW tumor, the Thorib® titanium bars system was fixed on both sides of the former rib with specific staples. Fixation on the sternocostal cartilage was avoided because of the high risk of early disjunction. If the tumor location did not allow the fixation on a bone structure, bars could be secured on the other side of the CW. For postero-lateral CW tumors when posterior fixation was impossible, the Thorib® bar was reshaped in a Z shape and fixed on the upper and lower rib (Figures 2, 3).

##### 2.2.2.2 Sternal reconstruction

Trionyx® sternal plate was used for large sternal defects (>10 cm) after sub-total or total sternectomy. The Trionyx® system is made of a central plate with arms that were fixed to two or three ribs on each side of the CW. In case of partial sternectomy, such as manubriectomy, one or two Thorib® bars were inserted from each side of the CW on the second or third rib.

##### 2.2.2.3 Mesh and flap

Muscular or musculocutaneous flaps were performed when there was a history of thoracic radiation therapy or when there was a large associated soft tissue resection. Pectoralis major muscle with skin advancement was mostly used for sternal resection and latissimus dorsi pedicled flap was the favorite choice for lateral rib resection. If a pedicled flap was not possible, a free flap was then performed. These procedures were performed in collaboration with the oncologic plastic surgery team. A resorbable Vicryl® (Ethicon, NJ, USA) mesh was used to protect the lungs from any damage from titanium devices.

##### 2.2.2.4 Sequence

The reconstructing sequence was performed as follows: chest drain insertion, Vicryl® mesh suture, titanium bar insertion, and flap covering.

### 2.3 Neoadjuvant and adjuvant treatments

Neoadjuvant treatment was decided by a specific tumor board following the national recommendations. Similarly, adjuvant therapy was decided by a specific oncologic tumor board, depending on the pathology, the extent of the disease, the patient's comorbidities, and the postoperative course.

### 2.4 Complications, operative mortality, survival, and follow-up

Postoperative complications were classified according to the Clavien–Dindo classification (15). More specifically, major pulmonary complication was defined as an absence of extubation or a re-intubation regardless of the cause. Minor pulmonary complications included lung congestion, pneumonia, atelectasis, non-severe pulmonary embolism, and the use of non-invasive ventilation. Diagnosis of surgical site infection is based on a body of clinical, biological, and chronological arguments: purulent flow from the scar or the chest tube, presence of germs in various sampling, or local inflammation; and compatible time of occurrence <1 year for early infection. Late infection was considered 1 year after the surgery. Chronic pain was defined when the patient presented with chest pain (using the Verbal Rating Scale) and has taken pain killer for 3 months or more. Implant failure was diagnosed if at least one of the following criteria was present: (1) bar or plate's fracture; (2) staple connector's unsealing; and (3) rib fracture next to the bar. Titanium allergy was based on fever, rash, erythema, effusion, granuloma, and hypereosinophilia without running infection.

Operative mortality was defined as death within the first 30 days after surgery or during the same hospital stay. Survival was calculated from the date of surgery until death or the date of last follow-up. Disease-free survival was calculated from the date of surgery until recurrence or the date of last follow-up. No patients were lost in the follow-up. Follow-up imaging studies consisted of routine chest X-ray, chest CTs, and PET/CT scans.

### 2.5 Statistical analysis

Statistical analysis was performed using GraphPad Prism Version 8.4.3 (471) (GraphPad Software, San Diego, CA, USA). Categorical variables were analyzed using the *chi*-square test, or Fisher's exact test for small samples. Continuous data were compared using Student's test (parametric test) or Mann–Whitney's test (non-parametric test). Overall survival rates were calculated using the Kaplan–Meier method, and a log-rank test was used to calculate survival differences between the two groups. Statistical significance was accepted as a *p-*value < 0.05.

## 3 Results

### 3.1 Patients' characteristics

Between June 2012 and December 2018, we identified 87 patients who underwent CW tumors resection and repair using Thorib® bars or Trionyx® sternal plate in our department. Nineteen patients were excluded (benign tumor, Pancoast Tobias tumor, palliative surgery, or clavicle fixation). Therefore, CW resection and reconstruction for malignancies were performed in 68 patients (26 men and 45 women), with a mean age of 56.4 years (range 18–80). Induction chemotherapy was performed in 20 patients, induction radiation therapy was performed in one patient and two patients had both. There were 29 sarcomas (including ten radiation-induced), 20 isolate CW metastasis, eight NSCLC, four breast cancer, three thymic malignancies invading the CW, and two localized sarcomatoid mesothelioma and one desmoid tumor. Patient characteristics data are summarized in Table 1.

**Table 1:** Patients’ characteristics.

	All		Thorib		Trionyx	
*n*	68		51		17	
Age						
Median, range	56.5	18–80	57.3	18–80	50	18–80
Follow-up						
Mean (months, range, SD)	43.2	(2.9–100.1); 30	40.3	(2.9–100.1); 31.0	52	(11.1–93.7); 27.2
Gender [*n* (%)]						
Male	26	38%	21	41%	5	29%
Female	42	62%	30	59%	12	71%
Co-morbidity						
BMI (mean) (kg/m^2^)	24.8		24.1		27	
Diabetes [*n* (%)]	5	7%	4	8%	1	6%
Smoking history [*n* (%)]	25	37%	20	39%	5	29%
Pack-year (mean)	9.8	(0–60)	11.5	(0–60)	5	(0–30)
Preoperative FVC (%, range)	101	(62–144)	102	(62–144)	99	(68–138)
Preoperative FEV1 (%, range)	96	(58–140)	96	(53–140)	96	(66–132)
Pathology						
Primary tumor [*n* (%)]	48	71%	42	82%	6	35%
Sarcoma	30	44%	26	51%	4	24%
Lung carcinoma	8	12%	8	16%	0	0%
Breast carcinoma	4	6%	3	6%	1	6%
Thymoma or thymic carcinoma	3	4%	2	4%	1	6%
Sarcomatoid mesothelioma	2	3%	2	4%	0	0%
Desmoid tumor	1	1%	1	2%	0	0%
Metastatic tumors [*n* (%)]	20	29%	9	18%	11	65%
Breast	12	18%	5	10%	7	41%
Sarcoma	3	4%	2	4%	1	6%
Colon	1	1%	1	2%	0	0%
Liver	1	1%	0	0%	1	6%
Larynx	1	1%	1	2%	0	0%
Parotid	1	1%	0	0%	1	6%
Kidney	1	1%	0	0%	1	6%

BMI, body mass index; FVC, forced vital capacity; FEV1, first second of forced expiration.

### 3.2 Surgical results

We used the Thorib® bars system in 51 patients with a mean of 1.35 bars per patient (range 1–3). The mean number of resected ribs was 2.65 (range 1–7). For the Thorib® system, tumor location was sternal in 12/51 (23%), anterior and anterolateral in 29/51 (57%), strictly lateral in 6/51 (12%), and postero-lateral in 4/51 (8%). The flow chart is available in Figure 1. Among Thorib® patients, fourteen had a partial sternal resection. For larger sternal resection, Trionyx® system was preferred in seventeen patients.

Musculocutaneous flap was performed in 63% of the patient (43/68). Soft tissue coverage of thoracic defect was pectoralis major muscle with skin advancement in eleven patients, musculocutaneous latissimus dorsi pedicled flap in 27 patients, or free musculocutaneous flap in six patients [deep internal epigastric perforating (DIEP) free flap or controlateral latissimus dorsi flap]. No omentoplasty was performed. Associated organs resections consisted of wedge resection (*n* = 11), lobectomy (*n* = 10), mastectomy (*n* = 10), thymectomy (*n* = 5), pericardium (*n* = 3), vessels (*n* = 2), phrenic nerve (*n* = 1), diaphragm (*n* = 1).

The definitive pathology examination showed complete R0 resection in 64 patients (94%) patients and R1 resection in four patients (6%). Two patients initially had an incomplete resection and had an early surgical revision with clean margins.

There were no per-operative and in-hospitality deaths. There were 66/68 patients (97%) extubated within the 24 first hours. The median length of postoperative care unit and hospital stays were 1 day (range 0–4) and 11.5 days (range 4–112), respectively. For patients who required intensive care unit, the median length of stay was 7 days (range 2–100). Forty-nine percent of the patient experienced a post-operative complication more than grade I from the Clavien–Dindo classification. Among them, sixteen had a grade II (24%), two had a grade IIIA (3%), eight had a grade IIIB (12%), five had a grade IVA (7%), and two had a grade IVB (3%).

### 3.3 Specific bar's and plates complications

No patient experienced a paradoxical chest movement although respiratory failure occurred in six patients (9%) (Table 2).

**Table 2:** Early and late outcomes after chest wall reconstruction using titanium.

	All		Thorib		Trionyx		*p-*Value
*n*	68		51		17		
Infection	12	18%	9	18%	3	18%	1
Early	11	16%	8	16%	3	18%	1
Late	1	1%	1	2%	0	0%	1
Implant failure	4	6%	3	6%	1	6%	1
Chronic pain	16	24%	10	20%	6	35%	0.20
Titanium allergy	0	0%	0	0%	0	0%	1
Respiratory failure	6	9%	6	12%	0	0%	0.32
Bar or plate removal	7	10%	7	14%	0	0%	0.18
Reoperation without removal of the bars or plate	6	9%	3	6%	3	18%	0.16

Infection of the operative site occurred in twelve patients (18%). Most of them had an early infection, but they required removal of the bars only in 50% of the cases (six patients). Others had also surgery with washing of the operative site without removing bars or plates. No Trionyx® system had to be removed for the three infections. No seroma was detected around the bars and plates during the follow-up. One patient had a late infection (more than a year after the initial surgery), due to the skin atrophy of the muscular flap and Thorib® bar exposure. Only the exposed bar had been successfully removed.

Implant failure occurred in four patients (6%) (three patients with Thorib® and one with Trionyx®). The patient with the Trionyx® system had an asymptomatic costal unsealing found in a CT during the follow-up. Only one patient with Thorib® failure needed a reintervention almost 4 years after the initial surgery. Another patient had an asymptomatic connector unsealing 8 years after the initial surgery, but due to the age of the patient and the asymptomatic fracture, we decided not to re-operate. The last patient had an implant failure associated with infection and loco-regional recurrence and has not been re-operated.

Chronic pain was the most common complication and occurred in 24% (16/68). The incidence did not differ between Trionyx® and Thorib® systems (35% vs. 20%, *p* = 0.22). A sensation of corset was also reported by these patients.

We did not diagnose any titanium during the follow-up.

### 3.4 Oncological outcomes

**Figure 4:**
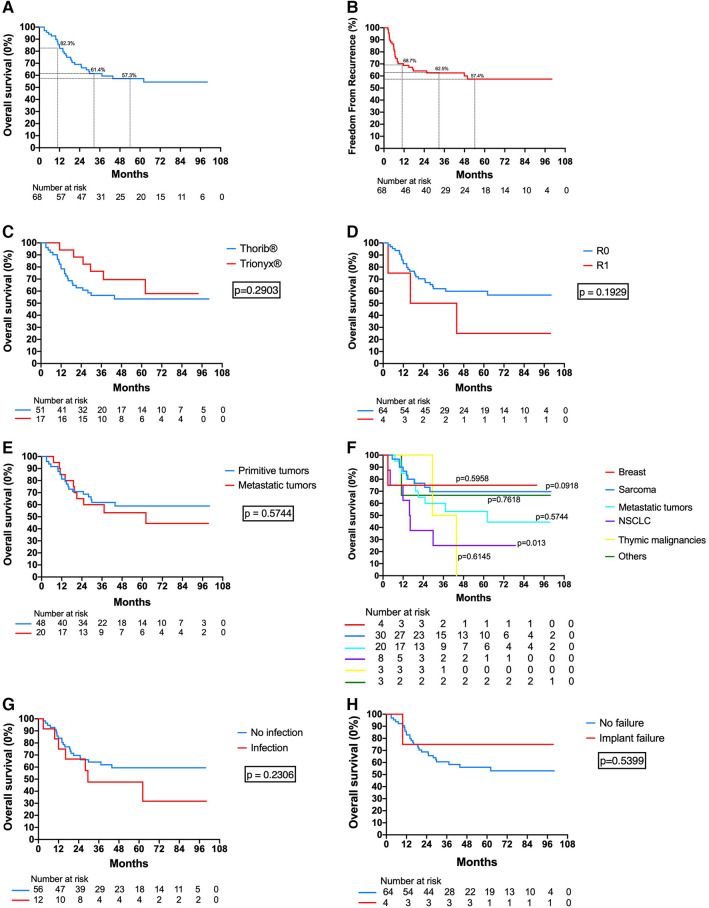
Survival and recurrence of the study population: (**A**) Overall survival of the whole cohort. (**B**) Freedom from recurrence of the whole cohort. (**C**) Overall survival according to the type of titanium reconstruction (Thorib® or Trionyx®). (**D**) Overall survival according to the completeness of resection. (**E**) Overall survival according to the primitive or metastatic nature of the tumor. (**F**) Overall survival according to the tumor's histopathology. (**G**) Overall survival according to the presence of surgical site infection. (**H**) Overall survival according to the presence of implant failure.

The median follow-up duration was 34 months. The 1-year, 3-year, and 5-year overall survival rate and freedom from recurrence rate were 82.3%/61.4%/57.3% and 67.6%/57.3%/52.6%, respectively (Figures 4A,B). Median survival for the overall population was unreached. Twenty-nine patients died during the follow-up including 25 patients who died from their CW tumors. Two others died from another cancer, one from an infection, and one from an unknown cause. No difference in survival was found regarding the material used (Thorib® vs. Trionyx®, *p* = 0.5688, Figure 4C), the completeness of resection (*p* = 0.1929, Figure 4D), the primitive or metastatic nature of the tumors (*p* = 0.5744, Figure 4E), and the occurrence of infection (*p* = 0.2306, Figure 4G) and implant failure (*p* = 0.5399, Figure 4H). Only NSCLC had a significantly worse survival than the other histopathology (*p* = 0.013, Figure 4F).

### 3.5 Long-term respiratory function

**Figure 5:**
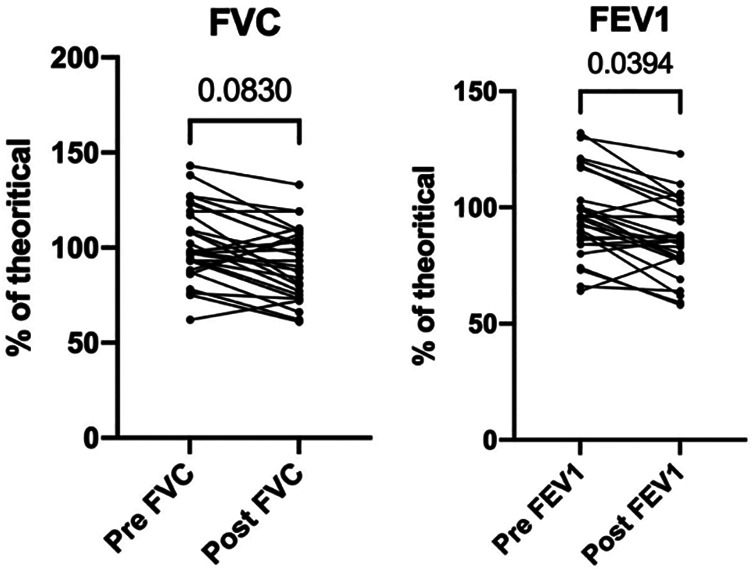
Long-term respiratory function before and after chest wall tumor resection and repair. (**A**) Forced vital capacity (FVC) before surgery and after surgery (≥3 months). (**B**) First second of forced expiration (FEV1) before surgery and after surgery (≥3 months).

Long-term pulmonary function tests were available in 29 patients among the 38 who were alive at the time of the study. Postoperative forced vital capacity (FVC) and the first second of forced expiration (FEV1) were 10% lower after CW resection and reconstruction (95% ± 17% vs. 85% ± 15%, *p* = 0.053; 102%±19% vs. 95%±18%, *p* = 0.03, respectively) (Figure 5).

## 4 Discussion

We demonstrated that CW reconstruction using titanium bar and plate could achieve satisfactory long-term results in terms of survival and complication rate. The primary aim of CW resection for cancer is to achieve a complete en-bloc resection*.* Titanium-based reconstruction allows thoracic surgeons to perform larger resections without increasing the risk of paradoxical motion with acute respiratory insufficiency. Titanium is an ideal prosthetic material, as it demonstrates high resistance to corrosion, low weight, remarkable resistance to traction forces, and high biocompatibility. CW reconstructions with titanium bars and plates have dramatically changed post-operative early outcomes. Paradoxical chest movement has become rarer, and patients are now facing long-term issues.

In the present study, we reported early and long-term results of a novel CW repair system called Thorib® for rib replacement and Trionyx® for sternal replacement. Our titanium system showed good results with non-paradoxical chest movement, and 97% of extubation on the day of surgery regardless of the size of chest resection. Titanium bars and plates allowed us to perform more extended surgeries, leading to a high rate of R0 resection. However, postoperative complications of such complex procedure remained relatively high with a total of 49% of grade II or more complications according to the Clavien–Dindo classification (15). Serious complications (grade III and IV) occurred in 25% of patients, but there was no postoperative mortality. Infection remained the main early complication whereas chronic pain remained the major long-term complication impacting the patient's quality of life.

Our 18% infection rate is higher than the previously published rate (6, 18). Considering the number of patients who received radiation therapy (radiation-induced sarcoma) and induction therapies, we assumed that our population of patients was at a higher risk of infection. Interestingly, we demonstrated that in case of surgical site infection, titanium removal was not mandatory. Specific interface design of titanium is known to make its surface less interactive (16). Berthet et al. even showed that titanium implants could be used in CW infection management (17). Importantly, revision surgery has always been performed to wash the material and the surgical site. In case decision of titanium removal has been made, surgical procedure did not request high skills and did not damage any surrounding structures. Implant infection rate is very variable in the literature, probably due to the diversity of materials used for CW reconstruction and the vague definition of surgical site infection in thoracic surgery (sometimes probably called “wound infection”). On a large retrospective cohort of 427 patients with CW reconstruction (19% of rigid reconstruction), Spicer et al. report 2.8% of wound infection (including 0.7% of empyema), with no explant due to infected mesh. On the other hand, a study using non-rigid prostheses by Lans et al. reported that 41 of the 220 patients (18.6%) with postoperative wound infection or wound necrosis after CW surgery. In studies with only sternal reconstruction with the STRATOS system, the surgical site infection rate was reported at 4%–5.6% (6, 18).

We reported a 6% rate of implant failure which could be considered as low compared to the literature. A bi-centric study found a 44% rate of failure with STRATOS™ and MatrixRIB® fixation systems (Synthes®, Solothurn, Switzerland) with a mean follow-up of less than 2 years (10). In this study, 70% of the failed implant had to be removed and failure was associated with the anterior location and multiples bars. Long-term results with the Synthes® sternal titanium system and MatrixRIB® fixation system reports three implant failure on 27 patients (11%) (7). Also, implant failure was frequently reported in the follow-up of patients with pectus excavatum correction (11, 19). In our study, the Thorib®/Trionyx® system seems to be very reliable provided fixation on the sternocostal cartilage is avoided.

We did not notice any titanium allergy, which confirms the biocompatibility of the Thorib®/Trionyx® bars and plates. Metal allergy is very rare and has been described mostly in orthopedic surgery (20). In the thoracic surgery field, metal allergy has been found in 2.2% of patients who underwent a Nuss procedure with a stainless steel bar over a period of 18 years (12). Even though titanium is recommended for metal allergy or history of metal allergy, titanium allergy can occur anyway (13). A history of metal allergy or atopy is frequently found in these patients (12, 13). Although metal allergy is rare, surgeons should keep in mind such complications, especially with the increasing use of titanium implants. Metal allergies are sometimes misdiagnosed as surgical site infections. It should be suspected in patients with a clinical presentation of fever, rash, erythema, effusion, and granuloma formation, without evidence of infection. A dermal test would be recommended before implantation if medical history of atopy (asthma, allergic rhinitis, eczema), allergies to jewelry, orthodontic braces, metal buttons/snaps on clothing, and food are discovered at the preoperative examination.

Chronic pain was the major complaint in patients with long-term survival (24%). Trionyx® plates seem to have a higher rate of chronic pain (35%) probably due to the number of resected ribs. Of importance, patients described the sensation of corset around the chest which could also explain any discomfort sensation. This sensation could be the consequence of the use of a rigid material or thoracic denervation. In 1983, LeRoux, one of the pioneers in the use of prostheses for CW reconstruction, described the characteristics of an ideal prosthetic material which should be sufficiently rigid to abolish paradox, biologically inert, tolerated and incorporated by the host, and able to be manipulated and shaped to fit a defect. Nowadays, paradox is no more an issue with titanium bars and plates, but a more flexible material may be able to reduce this sensation of CW rigidity.

We have no information about possible consequences in the case of cardio-pulmonary resuscitation using chest compression. The presence of bars behind the sternum like in the Nuss procedure notably lowered compression depth by a minimum of 69% compared to a chest without bar(s) (21). Except for Trionyx® bars attached from each side of the CW, titanium bars and plates would probably not be a contraindication for chest compression.

### 4.1 Strengths and limitations

This study was conducted at a single center and was retrospective. The low number of patients precluded any mortality, R1 resection, or functional outcomes statistical risk-factors analyses. However, CW tumors are rare diseases, and this report is the largest single-center series to date reporting long-term outcomes. Furthermore, CW malignancies were heterogeneous. At least, Thorib® bars have evolved in time, becoming more resistant for the second generation.

## 5 Conclusion

In summary, our study shows that Thorib® and Trionyx® bars and plates are safe and reliable in time, without paradoxical CW motion. Despite a high morbidity rate (especially surgical site infection) secondary to extended resection and post-radiation surgery, this new titanium system allowed the surgeons to perform more extended surgeries with a high rate of complete resections. Chronic pain is a long-term issue in ¼ of alive patients. CW resection and reconstruction with titanium bars achieved a 50% survival rate at 5 years for thoracic malignancies.

## Data Availability

The raw data supporting the conclusions of this article will be made available by the authors, without undue reservation.
